# Analysis of *Ginkgo biloba* Root Exudates and Inhibition of Soil Fungi by Flavonoids and Terpene Lactones

**DOI:** 10.3390/plants13152122

**Published:** 2024-08-01

**Authors:** Yawen Wang, Yanbing Jiang, Ximeng Liu, Yadi Chen, Qingxia Zhang, Li Wang, Weixing Li

**Affiliations:** 1 College of Horticulture and Landscape Architecture, Yangzhou University, Yangzhou 225000, China; yawenwang2003@163.com (Y.W.); 13205227999@163.com (Y.J.); luxiusl@163.com (X.L.); yadichen@yzu.edu.cn (Y.C.); liwang@yzu.edu.cn (L.W.); 2 College of Plant Protection, Yangzhou University, Yangzhou 225000, China; qxzhang@yzu.edu.cn

**Keywords:** *Ginkgo biloba*, secondary metabolites, root exudate, *Fusarium oxysporum*, *Rhizoctonia solani*

## Abstract

*Ginkgo biloba* is abundant in secondary metabolites, including flavonoids and terpenoids. While the majority of research has focused on the role of these compounds in disease resistance, their specific contribution to pathogen defense has been rarely explored. In this study, we collected root exudates from hydroponically cultivated ginkgo seedlings and conducted a metabolomic analysis. We identified several primary metabolites mainly comprising amino acids and nucleotides, while secondary metabolites consisted of various compounds, including bioactive compounds such as flavonoids and terpenoids. Focusing on the secondary metabolites with relatively higher abundance in the exudates, we selected a mixture of flavonoids and terpenoids for in vitro inhibition experiments against two soil-borne fungal pathogens, *Fusarium oxysporum* f. sp. *cucumerinum* that causes cucumber wilt and *Rhizoctonia solani* AG-8 that causes wheat root rot. The results indicated that the growth rate of both fungus cells was significantly reduced with the increasing concentration of the flavonoid and terpenoid mixture extracted from ginkgo and was completely inhibited at a concentration of 5 mg/mL. Further experiments revealed that this mixture of flavonoids and terpenoids had a destructive effect on the cellular structure of both fungi, thereby reducing cell viability and achieving an antifungal effect. These findings provide a foundation for further research into the use of ginkgo extracts in biological control.

## 1. Introduction

Root exudates act as a chemical conduit through which plants engage with soil microorganisms, utilizing an array of primary and secondary metabolites [1]. These compounds are emitted into the rhizosphere, where they mediate plant–microbe interactions and influence rhizosphere dynamics [1,2]. The release of root exudates occurs via three principal mechanisms: diffusion, ion channels, and vesicular transport. These exudates have the capacity to alter soil properties, serve as subsurface defensive agents, and mitigate sodium ion toxicity under saline conditions [3,4,5]. They also foster microbial acquisition of nutrients, leading to increased microbial biomass and enhanced root-associated activity [6]. Moreover, certain microbes assist plants in nutrient uptake and shield them from root pathogens [7]. For instance, low-molecular-weight sugars provide readily available carbon and nitrogen for microbes, while flavonoids can initiate signaling during mycorrhizal symbiosis [8]. Consequently, root exudates and microbes interact in a mutually beneficial manner, significantly impacting plant growth and adaptability.

Soil microorganisms are the primary constituents of soil ecosystem biomass and are crucial for sustaining subsurface ecosystem functionality [9,10]. This microbial community typically encompasses fungi, protists, viruses, bacteria, and archaea [11]. The diversity of soil microorganisms is generally shaped by climatic conditions, geographic location, and inter-microbe interactions [10,12,13]. While the beneficial roles of soil microorganisms have been extensively studied, they can also be a source of pathogens. Poor soil management and climate warming can disrupt microbial community structure, potentially leading to pathogen emergence [14,15]. For example, prolonged monoculture can alter peanut rhizosphere microbial communities, increasing the relative abundance of *Fusarium* and *Athelia*, which can cause infection and reduce crop yield and quality [14]. Similarly, in *Fragaria × ananassa*, serious fungal diseases caused by *Macrophomina phaseolina* are prevalent, leading to foliar wilt, senescence, stunting, and plant collapse [16]. However, in response to soil-borne diseases, root exudates can recruit beneficial microbes to combat pathogens. For example, after *Arabidopsis thaliana* is challenged by *Pseudomonas syringae*, the roots secrete malic acid, attracting *Bacillus subtilis* FB17 and activating the plant immune system [17]. Additionally, roots can secrete benzoxazinoids, which modulate root-associated microbial communities and promote jasmonate signaling for enhanced plant defense [18]. Overall, soil-borne diseases are prevalent, and exploring their link to root exudates is essential.

*G. biloba*, an ancient relict plant with a rich history, is one of the most studied plants. Its antifungal properties have garnered considerable research interest. *G. biloba* contains a variety of metabolites, including flavonoids, terpenoids and polyphenols, allyl phenols, and organic acids [19]. The seedcoat of *G. biloba* harbors ginkgolic acid, which exhibits antifungal activity against tubercle bacillus [20]. The leaves contain benzoic acid, a compound similar to ginkgo acid, which can inhibit the growth of various plant species [21]. Furthermore, *G. biloba* is a source of secondary metabolites such as ginkgolides A, B, C, D, and J found exclusively in leaves and ginkgolide M located only in the root bark of *G. biloba* [19]. Research has shown that *G. biloba* flavonoids possess antimicrobial effects against both Gram-positive and Gram-negative bacteria and fungi [22]. Moreover, the leaves are rich in polyphenols and flavonoids, which significantly inhibit *Staphylococcus aureus* [23]. However, there is a scarcity of research on the composition of *G. biloba* root exudates and the potential inhibitory effects of significant secondary metabolites from ginkgo on soil fungi.

In this study, we employed a hydroponic system to cultivate ginkgo seedlings, collecting root exudates for metabolomic analysis, which identified a plethora of primary and secondary metabolites, including the important secondary metabolites such as flavonoids and terpenoids present in ginkgo. Focusing on these two types of compounds and using the *G. biloba* leaf extract, which contains flavonoids and terpenoids as its main components, we further investigated their inhibitory effects on soil fungi, including *Fusarium oxysporum* f. sp. cucumerinum (Foc), the pathogen causing cucumber wilt, and *Rhizoctonia solani* AG-8 (*R. solani* AG-8), which causes wheat root rot. We found that a mixture of flavonoids and terpenoids from ginkgo effectively suppressed the growth of both fungi, and cytological observations unveiled the mechanisms underlying the suppression of fungal growth. This research provides a theoretical reference for the use of green bioactive substances to inhibit soil-borne diseases in agricultural production.

## 2. Materials and Methods

### 2.1. Experimental Material and Root Exudate Collection

Robust and uniform *G. biloba* seedlings that have been cultivated for 4 months in an artificial climate chamber under a 16 h light/8 h dark photoperiod at a temperature of 23 °C were selected. Soil from the root systems was gently removed using double-distilled water, and then, they continued to grow in double-distilled water with 10 seedlings per container, ensuring that the roots are shielded from light with a thin foil. 

Following a four-week incubation period, root exudates were collected and subjected to filtration and vacuum freeze-drying. The sample is to be taken in a volume of 9 mL and placed into the corresponding 50 mL centrifuge tube. It should be frozen in a −80 °C refrigerator overnight and then subjected to vacuum freeze drying. Following freeze-drying, 70% ethanol was added at a solid/liquid ratio of 30:1. After vortexing for 15 min, sonication in an ice water bath for 10 min (with the frequency of the ultrasonic instrument being 40 KHz, ultrasonic power being 80%), centrifugation at 12,000 rpm at 4 °C for 3 min, and filtration through a 0.22 μM filter membrane, the supernatant was collected and stored in an injection vial for subsequent LC-MS/MS analysis. These supernatants are stored at 4 °C before analysis.

### 2.2. Root Exudate Extraction and Metabolomic Analysis by UPLC-MS/MS

The metabolism of *G. biloba* root exudates was assessed using the non-targeted metabolome method at Metware Biotechnology Co., Ltd. (Wuhan, Hubei, China) with a recently reported method [24]. The root exudate data acquisition system comprised ultra-high liquid chromatography (ExionLC^TM^ AD, Framingham, MA, USA) and tandem mass spectrometry (Applied Biosystems 6500 QTRAP, Framingham, MA, USA). HPLC analyses were conducted on an Agilent SB-C18 (1.8 μm, 2.1 × 100 mm, Santa Clara, CA, USA) column. The mobile phase A consisted of ultrapure water (with 0.1% formic acid added), while the mobile phase B was acetonitrile (with 0.1% formic acid added). An injection volume of 2 μL was employed, with a flow rate of 0.35 mL/min. The proportion of phase B was 5% at 0.00 min, increased linearly to 95% within 90 min, and was maintained at 95% for 1 min. Thereafter, the proportion of phase B was reduced to 5% from 10.00 to 11.10 min and subsequently equilibrated at 5% up to 14 min. The Metware Database (MWDB) was employed to characterize the substances based on the secondary spectral information.

The ESI source operation parameters were as follows: the temperature of the electrospray ionization (ESI) source was set to 500 °C; the ion spray voltage (Ion Spray, IS) was 5500 V (positive ion mode)/−4500 V (negative ion mode); the ion source gas I (Gas1, GSI), gas II (Gas2, GSII), and curtain gas (Curtain Gas, CUR) were set to 50, 60, and 25 psi, respectively; and the collision-induced ionization parameter was set to high. Quantitative triple-quadrupole (QQQ) scans were conducted using the multiple reaction monitoring (MRM) mode, with the collision gas (nitrogen) set to medium. The declustering potential (DP) and collision energy (CE) of individual MRM ion pairs were optimized further. A specific set of MRM ion pairs was monitored in each period based on the metabolites eluted within that period. 

### 2.3. Inhibitory Efficacy on Fungal Growth

*G. biloba* leaves are a rich source of secondary metabolites. In addition to flavonoids and terpene lactones, the crude extract of ginkgo leaves also contains ginkgolic acids and other compounds, and our research revealed that the main compounds of these secretions were flavonoids and terpenoids. Due to the extraction and separation processes of *G. biloba* root exudates being relatively difficult, and the inconvenience of quantifying active compounds, therefore, we use commercial extracts to explore inhibitory efficacy on fungal growth. *R. solani* AG-8 was cultivated on potato dextrose agar (PDA) medium in a constant temperature incubator at 28 °C [25,26]. After culture about 5 d, a 5 mm hole was punched on the edge of the test strain. Fungal discs were then inoculated onto PDA medium containing different concentrations of ginkgo extract (Extract of *G. biloba* Leaves Drops, Kinnado) and cultivated at a temperature of 28 °C. Growth was monitored by taking post-inoculation photographs, and the inhibition rate of the fungi was calculated.

### 2.4. Detection of Membrane Permeability, Cell Viability, ROS Accumulation, Membrane Permeability, and Autophagy

A total of 1 mL mycelium that has been grown in liquid PDA medium for 5 d was centrifuged at 6000 rpm for 5 min to pellet the cells. The pellet was then gently resuspended in 200 μL of PBS (pH 7.4) buffer. After incubating with the dye, fluorescence microscopy (Axio Scope A1, Oberkochen, BW, Germany) was used to observe membrane permeability, cell viability, ROS accumulation, and autophagy. Membrane integrity was detected by 20 mg/L propidium iodide (PI), 10 μM 2,7-dichlorodihydrofluorescein diacetate (DCHF-DA) was used for examining ROS levels, 50 mg/L fluorescein diacetate (FDA) was used for detecting cell vitality, and 20 mg/L monodansylcadaverine (MDC) was used for the detection of autophagosomes. Before detecting fluorescence, the hyphae and the corresponding stains were incubated in the dark (for PI staining, the hyphae were incubated for 10 min; for DCHF-DA staining, the hyphae were incubated for 20 min; for FDA and MDC staining, the hyphae were incubated for 30 min separately). Subsequently, the hyphae were rinsed twice with PBS (pH 7.4), then re-suspended in 200 μL of PBS, and observed under a laser confocal microscope (ZEISS LSM 880, Oberkochen, BW, Germany) for fluorescence.

## 3. Results

### 3.1. Collection and Metabolic Profiling of Ginkgo Root Exudates

**Figure 1:**
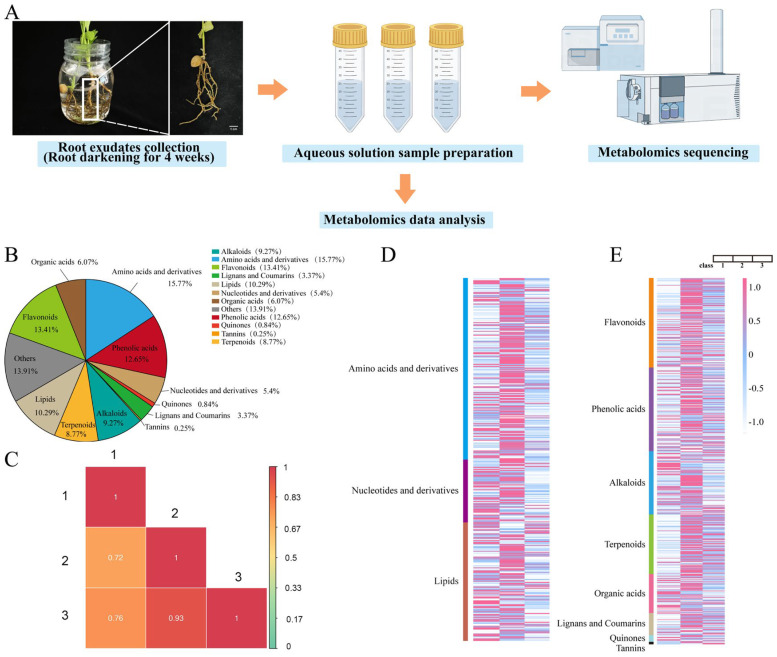
Collection and detection of ginkgo root exudates. (**A**): root growth of ginkgo seedlings cultivated in water; (**B**): collection of ginkgo root exudates; (**C**): the correlation analysis of the secretion of ginkgo root system, the redder the color, the higher the correlation coefficient, and the more reliable the obtained differential metabolites; (**D**): the heat map distribution of primary metabolites; and (**E**): the heat map distribution of secondary metabolites.

We utilized the hydroponic method to cultivate four-month-old ginkgo seedlings for a period of four weeks. After the cultivation, the root exudate water solution was subjected to metabolomic analysis (Figure 1A).

The metabolomic data showed that the ginkgo root exudates contain three major classes of substances: primary metabolites, secondary metabolites, and other substances. Among the primary metabolites, amino acids and nucleotides are the main components, especially amino acids, such as arginine, lysine, glutamine, and their derivatives, which account for as much as 15.77% of all detected metabolites. The secondary metabolites are divided into eight categories, with flavonoids, phenolic acids, and terpenoid lactones showing a significant advantage in the exudates, with flavonoids being the most abundant, accounting for 13.41%. In contrast, the proportion of tannin compounds in the exudates is relatively low, at only 0.25% (Figure 1B). To ensure the reliability of the data, each sample was independently replicated three times, and the positive correlation between the data was confirmed through Pearson Correlation Analysis (PCA), thereby verifying the validity and reproducibility of the metabolomic data (Figure 1C).

Additionally, primary metabolites, particularly amino acids and their derivatives, as well as nucleotides and their derivatives, were found in high concentrations in the secretion of ginkgo root exudates in these three sets of replicate data (Figure 1D). Furthermore, the eight various secondary metabolites also consisted of distinct components (Figure 1E), illustrating the variety of components in the ginkgo root exudates.

### 3.2. Component Analysis of Secondary Metabolites in Ginkgo Root Exudates

In the classification of secondary metabolites from ginkgo root exudates, our identification results showed a rich diversity of compounds, including flavonoids, phenolic acids, terpenoids, alkaloids, tannins, quinones, lignans, coumarins, and organic acids. Secondary metabolites exhibit a remarkable diversity, with each class encompassing a multitude of distinct chemical entities, which makes them highly complex in terms of chemical composition. Among them, flavonoids in ginkgo were particularly diverse, with 159 different components identified. These include a variety of molecular structures such as 3-hydroxy-4′,5,7-trimethoxy-flavanone, 5-hydroxy-3-(2-hydroxy-benzyl)-7-methoxy-chromann-4-one,4,4′-dihydroxy-2-methoxy-chalcone,5,6,3′,4′-tetrahydroxy-3,7-di-methoxy-flavone, and 6-methyl-flavone. Another significant group of secondary metabolites is the terpenoids, which make up 8.77% of the root exudates. These include cafestol, curcumenol, ginkgolide B, and rhizolactone. 

It also includes phenolic acid compounds containing 150 components, including phthalic anhydride, 3, 4-dimethoxyphenylacetic acid, dimethyl phthalate, and so on. Lignans and coumarins contain 40 different chemical components, with representative substances being 7-(6′,7′-dihydroxygeranyloxyl) coumarin, daphnetin, and nine linic acid. Additionally, the composition of tannins is relatively straightforward, consisting of only three different chemical components, which include 3,3′-*O*-dimethyllauric acid, 3,3′,4-*O*-trimethyllauric acid, and proanthocyanidin B1 (Table 1).

### 3.3. Effective Inhibition of Foc Growth by Flavonoids and Terpenoids from Ginkgo Extract

Given that ginkgo root secretions contain significant secondary metabolites such as flavonoids and terpene lactones, we utilized a commercially available ginkgo extract for our antifungal experiments. This extract contains a concentration of 9.6 mg/mL of total flavonol glycosides and 2.4 mg/mL of terpenoids. After dissolving the extract in a potato dextrose agar medium, it was diluted to various multiples for the bacteriostatic tests.

**Figure 2:**
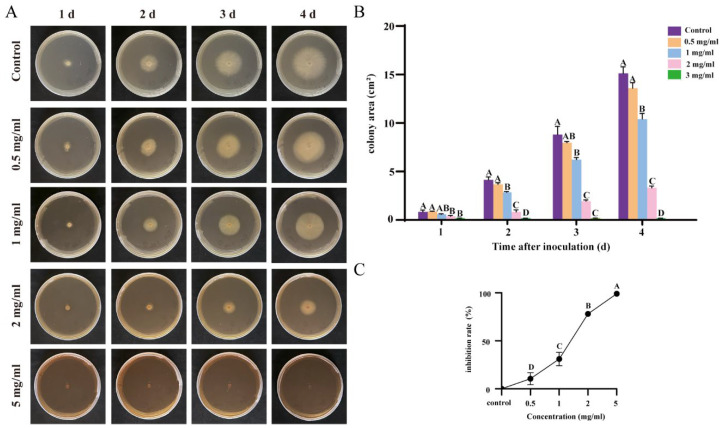
Inhibitory effect of different concentrations of *G. biloba* extracts on cucumber wilt disease caused by Foc. (**A**): Phenotypic observation of inhibition of growth of Foc by different concentrations of extracts. (**B**): Changes in area of Foc by different concentrations of extracts. Different capital letters indicate significant difference (*p* < 0.05). (**C**): Inhibition rate of different concentrations of extracts.

To explore the bacteriostatic effects of the flavonoids and terpenoids, we selected two prevalent soil-borne pathogens as study subjects. The cucumber-specific pathogen, Foc, a member of the subphylum *Pezizomycotina*, causes wilt in cucumbers, leading to symptoms such as leaf wilting and plant desiccation [25] (Xu et al., 2022). We found that as the concentration of the ginkgo extract increased, there was a notable reduction in the colony size of Foc on the medium. The growth of the pathogen was completely inhibited when the extract concentration was elevated to 5 mg/mL (Figure 2A). In contrast, in the control group without the extract and in the medium with a lower concentration of the extract, the colony area expanded over time (Figure 2B). Through multiple rounds of experimentation, it was confirmed that, at a concentration of 5 mg/mL, the *G. biloba* extract achieved an inhibition rate of 98.7% against Foc, substantiating its significant bactericidal impact on the pathogen (Figure 2C).

### 3.4. Flavonoids and Terpenoids from Ginkgo Effectively Inhibit R. solani AG-8

**Figure 3:**
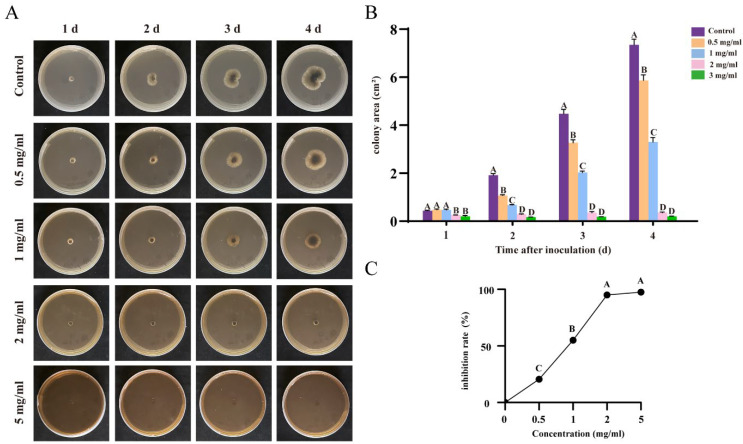
Inhibitory effect of different concentrations of ginkgo extracts on root rot of wheat caused by *R. solani* AG-8. (**A**): Changes in area of *R. solani* AG-8 by different concentrations of extracts. (**B**): Inhibition rate of different concentrations of extracts. Different capital letters indicate significant difference (*p* < 0.05). (**C**) Inhibition rate of different concentrations of extracts.

Wheat root rot is a fungal disease caused by *R. solani* AG-8, which presents symptoms similar to root retting and is closely associated with specific climatic conditions. This pathogen causes the wheat roots to turn black and decay [26] We found that, as the duration of treatment with ginkgo extract increased, the colony area of the root rot fungus expanded on the medium. However, an increase in the concentration of the extract led to a significant deceleration in the growth rate of *R. solani* AG-8. Notably, when the extract concentration was elevated to 2 mg/mL, the growth of *R. solani* AG-8 was markedly inhibited, with minimal mycelial formation observed. Upon further increasing the concentration to 5 mg/mL, the fungal growth activity nearly ceased, and no new colonies were formed (Figure 3A). The *G. biloba* extract exerts a pronounced inhibitory effect on *R. solani* AG-8, with the effect intensifying as the concentration increases. At a concentration of 5 mg/mL, the extract achieves an inhibitory rate of 97.4% against *R. solani* AG-8 (Figure 3B,C).

### 3.5. ROS Accumulation and Autophagy Activity in Foc and R. solani

**Figure 4:**
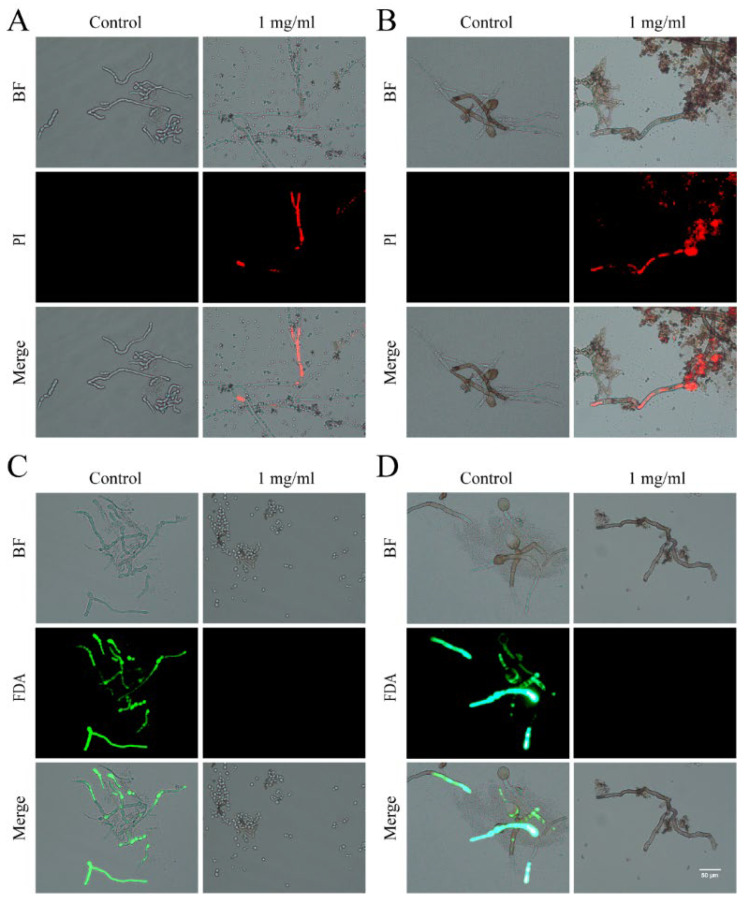
Ginkgo extract reduces Foc and *R. solani* AG-8 viability and disrupts membrane integrity. (**A**): Foc stained by PI; (**B**): *R. solani* AG-8 stained by PI; (**C**): Foc stained by FDA; (**D**): *R. solani* AG-8 stained by FDA.

Propyl iodide (PI) is a fluorescent dye that is commonly utilized for nuclear staining through broken cell membranes and is commonly used to detect apoptosis [27]. Our application of PI staining showed minimal fluorescence in the control group, whereas the majority of cells from Foc and *R. solani* AG-8 exhibited red fluorescence after treatment with the ginkgo extract, indicating that cell membrane integrity had been compromised (Figure 4A,B). This suggests that a high concentration of ginkgo extract can cause damage to the fungal cell membrane. Furthermore, fluorescein diacetate (FDA), which is used to assess the viability of plant and animal cells as well as plant cell protoplasts [28], preferentially stained the control group green. This indicates that the untreated control group cells experienced minimal damage and demonstrated higher cell viability (Figure 4C,D). In contrast, high concentrations of the ginkgo extract led to *R. solani* AG-8 damage and a decrease in cell viability.

### 3.6. Flavonoids and Terpenoids from Ginkgo Enhance the Levels of ROS and Autophagy Activity in Foc and R. solani AG-8

**Figure 5:**
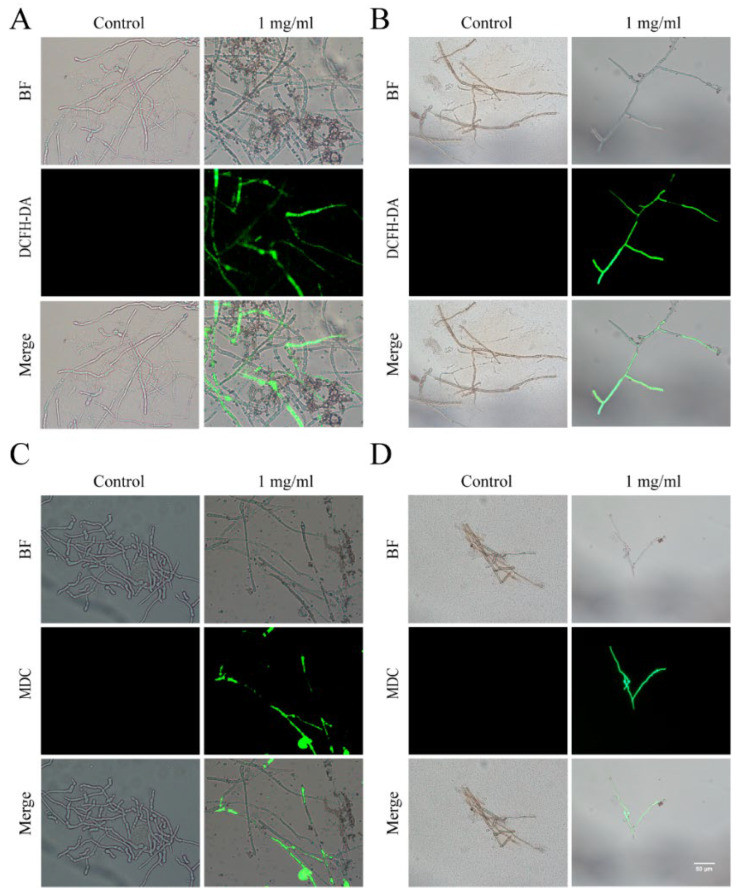
Ginkgo extract improves Foc and *R. solani* AG-8 ROS levels and autophagic activity. (**A**): Foc stained by DCFH-DA; (**B**): *R. solani* AG-8 stained by DCFH-DA; (**C**): Foc stained by MDC; (**D**): *R. solani* AG-8 stained by MDC.

DCFH-DA (dichlorodihydrofluorescein diacetate), a universal oxidative stress indicator, is frequently used to track cellular redox processes [29]. Our DCFH-DA staining showed no ROS accumulation in the control group. In contrast, significant ROS accumulation was observed in cells of Foc and *R. solani* AG-8 rot following treatment with the ginkgo extract (Figure 5A,B). MDC (monodansylcadaverine), a lysosomotropic fluorescent dye, is often used to detect autophagosome formation [30]. Our autophagosome detection with MDC showed negligible fluorescence in the control group, whereas the cells of the two fungi treated with the ginkgo extract displayed pronounced fluorescence (Figure 5C,D). These findings indicate that the treatment with ginkgo extract led to an accumulation of reactive oxygen species and an increase in autophagy activity in the cells of Foc and *R. solani* AG-8, thereby damaging the cells and inhibiting their growth.

## 4. Discussion

### 4.1. Ginkgo Root Exudates

*G. biloba*, a remarkable species with a long evolutionary history, has developed a complex chemical arsenal to deal with various environmental stresses. Flavonoids and terpene trilactones are among its chemical components, which are bioactive compounds that not only give ginkgo unique medicinal properties but also help the plant withstand different biotic and abiotic stresses [31,32].

Flavonoids, a class of secondary metabolites found widely in plants, have a variety of biological functions, including antioxidant, anti-inflammatory, antiviral, and immunomodulatory activities. In *G. biloba*, flavonoids such as quercetin, kaempferol, and isorhamnetin are present in the form of glycoside derivatives and aglycones [33]. These flavonoids contribute to the plant’s stress resistance by scavenging free radicals, inhibiting oxidative stress responses, and protecting plant cells from damage [34,35]. Furthermore, flavonoids may be involved in modulating plant signaling pathways, affecting growth and development, and enabling the plant to adapt to changing environmental conditions [36].

The terpenoids found in *G. biloba*, primarily synthesized in the root tissues and including ginkgolides and bilobalide [37], constitute another class of specialized metabolites in *G. biloba*. These compounds are thought to be involved in the plant’s response to pathogen attacks and other forms of stress [38]. For instance, they might participate in the plant’s defense against pathogens by inhibiting their growth or disrupting their metabolism, thereby protecting the plant [39]. Additionally, terpenoids could influence the plant’s photosynthesis and respiration, helping to maintain energy balance under stress [40].

In our study, we have identified a variety of flavonoids and terpenoids in the root exudates. Among the flavonoids present, a range of compounds has been detected, with some, such as sciadopitysin and echinatin, being particularly notable for their potential biological activities. Additionally, ginkgolide B stands out as a significant constituent that has been subject to extensive research due to its diverse pharmacological effects. Based on the identified components secreted, we hypothesize that these substances may exhibit certain resistance against soil-borne pathogens.

### 4.2. Significant Secondary Metabolites in Ginkgo Extract Effectively Inhibit Soil-Borne Fungi

*G. biloba* is rich in secondary metabolites. Current research primarily focuses on the synthesis of secondary metabolites in ginkgo leaves and their inhibitory effects on pathogenic microorganisms [19]. However, the composition of its root exudates and their effects on soil-borne diseases remain unclear. Our research has revealed that the main components of ginkgo root exudates are primary secondary metabolites such as flavonoids and terpenoids. Due to the complex and difficult to separate chemical composition of root exudates, we used extracts from ginkgo leaves (which mainly consist of flavonoids and terpenoids) to assess their inhibitory effects on cucumber wilt fungal and wheat root rot fungal, because the main components of root exudates are flavonoids and terpenoids. The ginkgo extracts are known to contain these bioactive compounds, with previous research highlighting the antifungal activity of flavonoids purified from ginkgo against food-borne pathogens. Specifically, quercetin, kaempferol, and isorhamnetin have been identified as the main contributors to this activity, operating through mechanisms that induce oxidative damage and cell wall and membrane disruption and enhance membrane permeability in bacteria [41].

Given the secretion of both primary and secondary metabolites such as flavonoids and terpenoids by the ginkgo root system, our study focuses on the use of widely available and cost-effective ginkgo crude extracts to investigate common soil-borne diseases. However, the majority of research has concentrated on the inhibitory effects of ginkgo extracts on pathogenic microorganisms in both animals and humans [42,43,44]. Studies have revealed significant antimicrobial activity of ginkgo extracts against a variety of bacterial species, with the flavonoid glycosides, quercetin, kaempferol, and isorhamnetin, showing notable inhibitory effects on both Gram-positive and Gram-negative bacteria as well as fungi [22]. Furthermore, *Escherichia coli*, *Klebsiella pneumoniae*, and *Yersinia enterocolitica* have been reported to be highly susceptible to ginkgo extracts, with *S. aureus* demonstrating the lowest MIC values, indicating it was the most affected by the extracts [23]. These studies underscore the potential of integrating ginkgo extracts into strategies aimed at combating microbial infections, due to their broad-spectrum antimicrobial activity against both Gram-negative and Gram-positive bacteria.

In our study, we selected Foc, which causes wilt disease in cucumbers, and *R. solani* AG-8, responsible for root rot in wheat, to evaluate the effects of ginkgo extracts containing flavonoids and terpenoids. Our findings indicated that these extracts could significantly inhibit the growth of Foc and *R. solani* AG-8, and with the increasing concentrations of the extracts, Foc and *R. solani* AG-8 could be completely suppressed. Cytological experiments further demonstrated that high concentrations of ginkgo extracts can damage the fungal cell membrane, leading to a decline in cell viability. Additionally, it was found that high concentrations of flavonoids can trigger autophagy in fungal cells, further reducing their activity.

The present study demonstrates the pronounced inhibitory effects of secondary metabolites, including flavonoids and terpenoids derived from ginkgo, on soil-borne fungi. However, it should be noted that the ginkgo extracts utilized in this study do not fully represent the comprehensive profile of root exudates. Nevertheless, our findings provide a rationale for considering ginkgo root exudates as a potential source of antifungal agents. Therefore, further experimentation may include the implementation of strategies such as crop rotation and intercropping, which are essential for validating the potential of these natural compounds in agricultural settings.

## Figures and Tables

**Table 1:** Types and components of secondary metabolites in ginkgo root exudates.

Category	Compounds	Number
Flavonoids	3-Hydroxy-4′,5,7-Trimethoxyflavanone; Texasin; 5-Hydroxy-3-(2-hydroxybenzyl)-7-methoxychroman-4-one; Kayaflavone*; 4,4′-Dihydroxy-2-methoxychalcone; Echinatin; Sciadopitysin*; 5-hydroxy-7-(1′,2′-dihydroxypropyl)-2-methyl-chromone; 3,7,4′-Trihydroxyflavone; 5,6,3′,4′-Tetrahydroxy-3,7-dimethoxyflavone; 6-Methylflavone	159
Phenolic acids	Phthalic anhydride; 3,4-Dimethoxyphenyl acetic acid; Ethyl 2,4-dihydroxy-3,6-dimethylbenzoate; 3-Hydroxycinnamic Acid*; 2-Hydroxycinnamic acid*; 4-Nitrophenol; Dimethyl phthalate; 6-*O*-Feruloyl-β-D-glucose; 4′-Methoxyacetophenone; Ethyl phenylacetate	150
Quinones	2-Hydroxy-2,3-dihydronaphthalene-1,4-dione; Danthron; Madeirin; 1,8-Dihydroxyanthraquinone; Hircinol; 8-Hydroxy-2-methoxy-1,4-naphthoquinone; Torachrysone-8-*O*-glucoside; Aurantio-obtusin-6-*O*-Glucoside*; 3-Methoxyjuglone; Anthraquinone-2-carboxylic acid; Chrysophanol-9-anthrone; 3-Hydroxydehydroiso-α-Lapachone;	10
Lignans and Coumarins	Marmin [7-(6′,7′-Dihydroxygeranyloxy)coumarin]; Daphnetin; 1′,2′-epoxy-4-isobutyryl coniferol; (7-Hydroxy-6-methoxycoumarin)*; 6-Hydroxy-7-methoxycoumarin*; Paniculin; Scopoletin; 6-Hydroxy-7-methoxycoumarin*; Sesaminol*; 6-(2′,3′-dihydroxy-3-methylbutyl)-8-prenyl umbelliferone; 4-HydroxyseSamin*	40
Tannins	3,3′-*O*-Dimethylellagic Acid; 3,3′,4-*O*-Trimethylellagic acid; Procyanidin B1	3
Alkaloids	Hordenine; Pseudoephedrine; Octadec-8-enamide; octadecadienoic acid amide; Laurocapram; Choline; Acetohexamide; 8β-acetoxy-12β-hydroxy-lycopodine; 5-Hydroxyindole; Hydroquinine	110
Terpenoids	Cafestol; 12,13-Dehydrogeranylgeraniol; Curcumenol; ginkgolide B*; Procurcumenol; 14-deoxycoleon U; ginkgolide X; 5α,6α,7β,10β-11α,13-dihydro-4(15)-eudesmene-12,6-olide Tsoongiodendroonolide; eupatorinol	104
Organic acids	Malonic acid; 3-(3-hydroxy-4-methoxyphenyl)propanoic acid; trans-4-Methylene-2-octyl-5-oxotetrahydrofuran-3-carboxylic acid; Muconic acid; 2-Cyclohexyl-2-hydroxy-2-phenylacetic acid; 4-Tert-butylphenoxyacetic acid; 3-(2-Methoxyphenyl)propanoic acid; 3-Carboxy-4-methyl-5-propyl-2-furanpropionic acid; Azelaic acid; Methanesulfonic acid	72

Marking with an asterisk (*) represents that the substance has stereoisomers.

## Data Availability

The data supporting the findings of this study are available within the article.
